# Evaluating the Direct and Indirect Toxicity of Nine Insecticides on an Important Predatory Natural Enemy in Rice Fields

**DOI:** 10.3390/insects17020187

**Published:** 2026-02-10

**Authors:** Mubashar Hussain, Jiachun He, Qi Wei, Fengxiang Lai, Pinjun Wan, Qiang Fu

**Affiliations:** State Key Laboratory of Rice Biology and Breeding, China National Rice Research Institute, Hangzhou 311401, China; hmubashir853@gmail.com (M.H.); weiqi01@caas.cn (Q.W.); laifengxiang@caas.cn (F.L.); wanpinjun@caas.cn (P.W.)

**Keywords:** direct and indirect toxicity, insecticides, natural enemies, predatory activity, safety risks

## Abstract

Natural enemies play an important role in the integrated pest management (IPM) of rice crops. Chemical control is commonly used for pest management in rice; however, the compatibility between biological and chemical control within this system has not been thoroughly investigated. This study aimed to evaluate both the direct and indirect toxicity of nine insecticides—tetraniliprole, triflumezopyrim, chlorantraniliprole, pymetrozine, spinetoram, nitenpyram, imidacloprid, emamectin benzoate, and avermectin—against six important predators of rice pests: *Cyrtorhinus lividipennis*, *Paederus fuscipes*, *Ummeliata insecticeps*, *Tetragnatha maxillosa*, *Mendoza cancestrinnii*, and *Pardosa pseudoannulata* under laboratory conditions. The results indicated that tetraniliprole, triflumezopyrim, and chlorantraniliprole exhibited negligible direct toxicity (mortality < 30%) to all six predators and did not significantly affect their predatory activity. In contrast, spinetoram, avermectin, emamectin benzoate, nitenpyram, and imidacloprid showed high direct toxicity (mortality > 99%), significantly reduced predatory activity, and were classified as high to extremely high risk for *C. lividipennis*. Nitenpyram showed strong direct toxicity (mortality > 99%) to *P. fuscipes* and was categorized as high risk. Avermectin and emamectin benzoate exhibited high direct and indirect toxicity to all four spider species, significantly reducing predatory activity, and were graded from medium to extremely high risk. Spinetoram reduced predatory activity across all four spider species and exhibited direct toxic effects, posing a high risk to *U. insecticeps*. In conclusion, pymetrozine, spinetoram, nitenpyram, imidacloprid, emamectin benzoate, and avermectin exerted lethal or sublethal effects on all six predators. Conversely, tetraniliprole, triflumezopyrim, and chlorantraniliprole were regarded as safer insecticides for all six predators.

## Simple Summary

Natural enemies play a crucial role in suppressing insect pests in rice ecosystems, but their safety is often neglected during insecticide selection. This study compared the effects of nine commonly used insecticides on six important predatory natural enemies of rice pests. The results showed clear differences in insecticide safety. Tetraniliprole, triflumezopyrim, and chlorantraniliprole had minimal direct or indirect toxicity to predators, indicating good compatibility with biological control. In contrast, spinetoram, pymetrozine, nitenpyram, imidacloprid, emamectin benzoate, and avermectin caused high direct or indirect toxicity to all six predators, including green mirid bugs, rove beetles, and spiders. These findings highlight the importance of selecting insecticides that effectively control rice pests while conserving beneficial natural enemies.

## 1. Introduction

Rice (*Oryza sativa* L.) is a crucial cereal crop and serves as a staple food for nearly 3 billion individuals worldwide. In China, rice is a predominant cereal crop and constitutes a staple food source for approximately 65% of the population [1,2]. However, rice cultivation in China is adversely affected by a range of insect pests, including major species such as rice planthoppers, rice stem borers, and the rice leaf folder [3]. Furthermore, planthoppers destroy rice crops by damaging plant nutrients and spreading different viral pathogens [4]. Rice stem borer larvae live and feed inside the rice stem and can cause 3–95% damage to the rice crop [5,6]. Rice leaf folder larvae fold the leaves longitudinally and feed by scraping the green mesophyll tissue from within the folded leaves. As a result of leaf damage, the general vigor and photosynthetic capacity of rice plants are reduced, making affected plants more susceptible to bacterial and fungal infection [7,8].

Various integrated pest management (IPM) strategies are employed to mitigate insect pests. Major IPM strategies include cultural control, biological control, and chemical control [9]. Among these, chemical control remains a pivotal component of IPM due to its rapid action, high efficiency, ease of application, cost-effectiveness, and reliable efficacy against pests [10]. Nevertheless, the improper application of chemical pesticides often results in adverse consequences, such as the development of insecticide resistance [11]. Moreover, the application of chemical insecticides poses a significant risk to populations of beneficial arthropods [12]. However, the detrimental effects of these insecticides on natural enemy populations often remain overlooked [13].

Within the rice ecosystem, a diverse array of beneficial arthropods, referred to as natural enemies, plays a very important role in reducing damage caused by insect pests. These natural enemies are categorized into two distinct groups: predators and parasitoids. Predators in the rice ecosystem include spiders, beetles, and mirid bugs, whereas parasitoids are primarily represented by wasps belonging to the order Hymenoptera. Together, these natural enemies regulate insect pest populations and constitute essential components of (IPM) programs [3,14,15,16].

Currently, more than 1375 species of natural enemies of rice insect pests have been recorded in China, including 889 species of predatory natural enemies. Predators account for approximately 64.74% of all recorded natural enemies [17,18]. Among predators, *Cyrtorhinus lividipennis* is an important predator of rice planthoppers, including *Nilaparvata lugens* and *Sogatella furcifera*. In rice fields of Xiaoshan County, Zhejiang Province, approximately 39.1% of *N. lugens* eggs were predated by *C. lividipennis* in 1982, while in Shaxian County, Fujian Province, 40–50% of *S. furcifera* eggs were predated by this species. *Paederus fuscipes* is another significant predator of rice pests such as *N. lugens*, *S. furcifera*, *Nephotettix cincticeps*, *Chilo suppressalis*, and *Tryporyza incertulas*. Adult *P. fuscipes* can predate 3.7–10.1 third- to fifth-instar nymphs of *S. furcifera* and 2.0–3.6 third- to fifth-instar nymphs of *N. cincticeps* per day. Among spiders, *Ummeliata insecticeps* is an important natural enemy of rice aphids and the young larvae of *Cnaphalocrocis medinalis*, *C. suppressalis*, and *T. incertulas* [3,16]. *Pardosa pseudoannulata* is recognized as a key biological control agent in rice fields, regulating populations of major rice pests such as planthoppers and leafhoppers [15]. *Tetragnatha maxillosa* and *Mendoza cancestrinnii* spiders also play a significant role in controlling rice pests, including rice planthoppers, rice leaf roller, and stem borers [14].

In earlier years, insecticides such as carbofuran, deltamethrin, thiamethoxam, and triazophos were widely used to control major rice pests. However, these insecticides had highly negative impacts on populations of natural enemies, including both parasitoids and predators [19,20]. Thiamethoxam, while effective against rice pests such as rice planthoppers, is highly harmful to natural enemies, including spiders, *Paederus alfierii* Koch, and *C. lividipennis* [21,22,23]. Similarly, deltamethrin and triazophos are highly toxic to spiders and *C. lividipennis* [24]. Carbofuran significantly reduces predator populations in rice crops, including ladybird beetles, wolf spiders, carabid beetles, earwigs, green mirid bugs, and damselflies [20].

On average, approximately four insecticide applications are carried out to control pests during each rice cultivation period [25]. In China, farmers commonly rely on chemical control to suppress major rice pests, particularly sucking insects such as rice planthoppers. Among the most frequently used insecticides are imidacloprid, avermectin, and nitenpyram, which are known for their rapid action and high efficacy against these pests [26,27] while, for controlling leaf-feeding pests, such as the rice leaf folder, spinetoram is widely applied due to its strong activity against lepidopteran larvae [28].

Currently, novel long-acting insecticides are being employed for the management of rice pests. Tetraniliprole is a new phthalic acid diamide group having a unique chemical structure and showing excellent activity against a broad spectrum of lepidopteran pests [14]. Triflumezopyrim is used to control rice planthoppers, including brown planthoppers and white-backed planthoppers [29]. Chlorantraniliprole is a new systemic insecticide of the anthranilic diamide group with a unique and new mode of action. It is used to control rice stem borers [30]. These insecticides are reported to have high efficiency in controlling rice insect pests; however, their impact on predators of rice pests is poorly documented.

This research investigates the effects of nine widely utilized insecticides: chlorantraniliprole, triflumezopyrim, pymetrozine, imidacloprid, avermectin, emamectin benzoate, tetraniliprole, spinetoram, and nitenpyram against six important predators of rice pests in China: *C. lividipennis*, *P. fuscipes*, *U. insecticeps*, *T. maxillosa*, *M. cancestrinnii,* and *P. pseudoannulata*. The assessment employs indoor toxicity bioassays and predation behavior tests, aiming to provide informed recommendations for the judicious application of chemical insecticides in rice cultivation.

## 2. Materials and Methods

### 2.1. Predators and Prey

Predators: *Cyrtorhinus lividipennis* (Hemiptera: Miridae), *Paederus fuscipes* (Coleoptera: Staphylinidae), *Ummeliata insecticeps* (Araneae: Linyphiidae), *Tetragnatha maxillosa* (Araneae: Tetragnathidae), *Mendoza canestrinii* (Araneae: Salticidae), *Pardosa pseudoannulata* (Araneae: Lycosidae), and prey *Nilaparvata lugens* (Hemiptera: Delphacidae) were collected from China National Rice Research Institute (CNRRI) fields in Hangzhou, Zhejiang province, China. Predators and *N. lugens* were kept in a greenhouse that maintained a temperature of 27 ± 1 °C and a humidity of 70 ± 5% relative humidity under natural light. Predatory spiders were fed in small plastic cups individually. Each spider was provided 5–10 brown planthopper 2nd to 3rd instar nymphs every day and a water sponge. *C. lividipennis* and *P. fuscipes* were reared and fed on rice plants with brown planthopper 2nd to 3rd instar nymphs in a cage. All natural enemies were reared for 1–2 generations, and newly emerged adult females were selected for the experiment.

### 2.2. Insecticides

Nine insecticides were selected for this study, named as follows: Chlorantraniliprole, triflumezopyrim, pymetrozine, imidacloprid, avermectin, emamectin benzoate, tetraniliprole, spinetoram, and nitenpyram (Table 1). Insecticides were dissolved in water and used directly for contact bioassays.

### 2.3. Methodology for Bioassay

#### 2.3.1. Toxicity Bioassay of Insecticides to Predators

The toxicities of insecticides to *C. lividipennis* and *P. fuscipes* were determined in the laboratory using the stem dipping method as described by Zhu et al. [29] with slight changes. Adult *C. lividipennis* and *P. fuscipes* were used in the experiment. Insecticides were tested at the maximum recommended field doses to assess direct toxicity and compatibility with predatory natural enemies. For LC_50_ determination, a series of five to six insecticide doses was prepared, such that the lowest concentration caused mortality comparable to the control treatment, while the highest concentration resulted in 100% mortality. For *C. lividipennis*, ten female *Nilaparvata lugens* were placed on rice plant stems 48 h prior to the experiment to allow oviposition, and the resulting eggs served as a food source during the bioassay. The females were removed after 12 h of oviposition. Mortality observations were performed after 48 h for *C. lividipennis* based on the methodology by Sun et al. [21], while brown planthoppers (2nd to 3rd instar) were served as food for *P. fuscipes*. Rice seedlings (late tillering to booting stages) were excised into 10 cm segments with intact roots, and stems were immersed in each insecticide solution for 30 s. After immersion, seedlings were maintained at room temperature for 10–30 min to allow insecticide solutions (including water control) to dry and then transferred to plastic cages (6.5 cm diameter × 10.5 cm length). Seedling roots were covered with cotton moistened with water. Ten adults of predators (*C. lividipennis*, *P. fuscipes*) were then introduced into each plastic cage. After 48 h exposure, the numbers of dead and live predators were counted, and the mortality (%) was calculated for each insecticide. A set of 10 adults in a plastic cage was considered to be one replicate, and each treatment had three replicates. Observations were performed after 48 h for *P. fuscipes* based on the methodology by Zhu et al. [29].

An immersion test method was used to evaluate the effects of insecticides on spiders as described by Zhu et al., with slight modification [29]. Four spider species: *Ummeliata insecticeps*, *Tetragnatha maxillosa*, *Mendoza cancestrinnii,* and *Pardosa pseudoannulata* adults were selected for this study. For direct toxicity determination, insecticides were tested at the maximum recommended field doses. However, to calculate the LC_50_, a series of 5–6 different doses of the insecticide were utilized. Adult spiders were dipped in the insecticide solutions for 20 s and then placed on absorbent paper to remove the remaining solution. Individual spiders were then transferred into plastic cups containing a water sponge and food BPH nymphs. The numbers of dead and alive predatory spiders were counted after 96 h, and the mortality (%) was calculated for each insecticide by following statistical methods. A set of 10 adult spiders was considered as one replicate, and each treatment had three replicates. Spiders’ mortality data were recorded after 96 h as described by Zhu et al. [29].

#### 2.3.2. Indirect Effects of Insecticides on the Predatory Activity of Predators

Adults *C. lividipennis* and *P. fuscipes* were individually starved for 24 h in plastic cups containing a water-soaked sponge and covered with nylon mesh prior to the experiment. Insecticide solutions were prepared at the maximum recommended field doses. Three control treatments using tap water were included for each insecticide. Insecticides were applied to rice plants using the stem-dipping method and allowed to dry for 30 min. Each predator was then released onto the insecticide-treated plant for 48 h, after which it was transferred to a new, insecticide-free plant containing 30 brown planthoppers (2nd to 3rd instar). Prey consumption was recorded after 48 h. Each insecticide treatment was replicated three times.

During the investigation of spider predation rates, insecticides were applied to adult spiders using the “immersion test” described above. Three control treatments using tap water were prepared for each insecticide. After insecticide application, one predatory spider was introduced into a cylindrical plastic cup (10 cm in diameter and 28 cm in height) containing TN1 rice seedlings. To assess predatory activity, 30 brown planthoppers were provided as prey on the rice plants: fifth-instar nymphs to adults for *T. maxillosa*, *M. cancestrinnii*, and *P. pseudoannulata*, and first- to second-instar nymphs for *U. insecticeps*. Prey consumption was recorded after 48 h. Each insecticide treatment was replicated three times. Predation rate was calculated by the following formula:

Predation rate % = (Number of prey consumed/Total number of prey) × 100%

### 2.4. Statistical Analysis

All experimental data were processed using Data Processing System (DPS) version 17.1 software to calculate the median lethal concentration (LC_50_) and 95% confidence limits for each insecticide.

The toxicities of insecticides were classified as recommended by the International Organization for Biological Control (IOBC) [31]. Insecticides were divided into four categories. Category 1-Harmless: <30 mortality (%), category 2-Slightly harmful: 30–70 mortality (%), category 3-Moderately harmful: 80–99 mortality (%), and category 4-Harmful: >99 mortality (%).

The safety factor evaluation was based on the evaluation of toxicity testing against the natural enemy Trichogrammatids in pesticide registration environmental testing [32]. The safety factor Risk Quotient (RQ) is the ratio of the pesticide’s LC_50_ value to the maximum recommended field doses. A safety factor of 0.05 or less indicates an insecticide with an “Extremely high-risk level”, a safety factor greater than 0.05 but less than or equal to 0.5 indicates a “High-risk level”, a safety factor greater than 0.5 but less than or equal to 5 indicates a “Medium-risk level” and a safety factor greater than 5 indicates a “Low-risk level” insecticide.

Data on predatory activity were analyzed using one-way ANOVA followed by Duncan’s new multiple range test [33].

## 3. Results

### 3.1. Direct Toxicity of Insecticides to Predators

Direct toxicity of insecticides to predators (Table 2) reveals that spinetoram, avermectin, emamectin benzoate, nitenpyram, and imidacloprid, at the recommended field dose, showed 100% mortality to *C. lividipennis,* while others were harmless (mortality < 30%). For *Paederus fuscipes*: chlorantraniliprole, triflumezopyrim, pymetrozine, imidacloprid, avermectin, emamectin benzoate, tetraniliprole, and spinetoram showed <30% mortality, while nitenpyram showed 100% mortality after 48 h exposure, which indicates that only nitenpyram was harmful to *P. fuscipes*.

The results of insecticides’ direct toxicity to spiders (Table 3) reveal that chlorantraniliprole, triflumezopyrim, pymetrozine, imidacloprid, and tetraniliprole showed <30% mortality to all four species of spiders. Avermectin and emamectin benzoate showed high toxic effects to all spiders and caused 100% mortality, while spinetoram showed 100% mortality results to *U. insecticeps* but safety to the other three species of spiders after 96 h exposure time. Results indicate that avermectin and emamectin benzoate were harmful to all four species of spiders.

### 3.2. Effects of Insecticides on Predatory Activity

**Figure 1:**
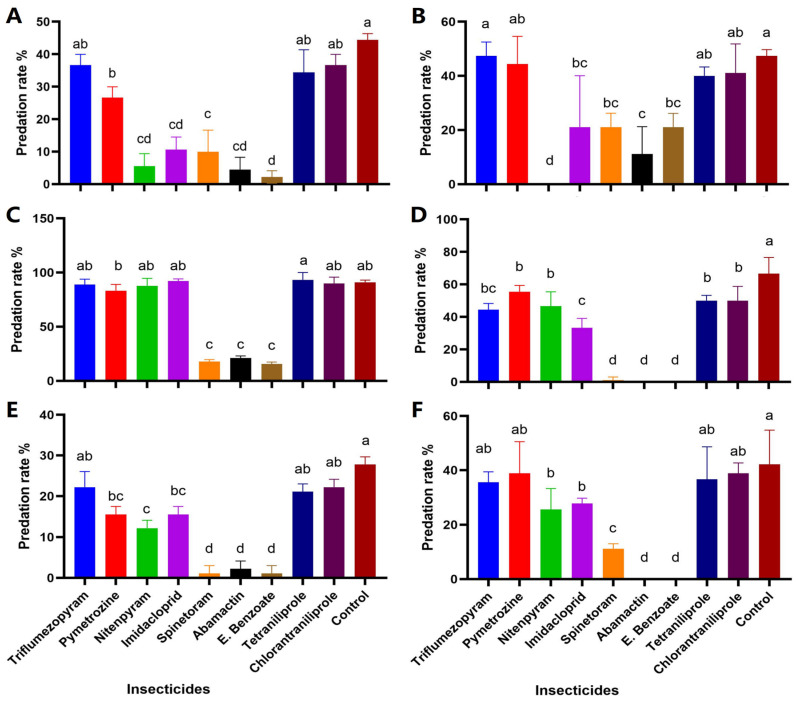
Impact of nine insecticides on predatory activity of predators: *Cyrtorhinus lividipennis* (**A**) *Paederus fuscipes*; (**B**) *Pardosa pseudoannulata*; (**C**) *Mendoza cancestrinnii*; (**D**) *Tetragnatha maxillosa*; (**E**) *Ummeliata insecticeps*; (**F**) Graphs represent (Mean ± SD) value for each insecticide. Different letters above bars indicate a significant difference at the 0.05 level by Duncan’s new multiple range test.

Exposure to different insecticides significantly affected the predatory activity of *C. lividipennis*. Nitenpyram, imidacloprid, pymetrozine, avermectin, emamectin benzoate, and spinetoram significantly decreased the predatory rate of *C. lividipennis,* while for the other three insecticides: tetraniliprole, triflumezopyrim, and chlorantraniliprole, there was no significant difference with the control treatment (df = 9/20, F = 21.70 and *p* < 0.0001) (Figure 1A). Among them, the predation rate is the lowest after treatment with emamectin benzoate. For *P. fuscipes*, the predatory rate has significantly decreased after treatment with nitenpyram, avermectin, imidacloprid, spinetoram, and emamectin benzoate. While tetraniliprole, triflumezopyrim, and chlorantraniliprole had no significant difference with control for *P. fuscipes* (df = 9/20, F = 8.69 and *p* < 0.0001) (Figure 1B).

*P. pseudoannulata* predatory activity results showed that by treatment of avermectin, emamectin benzoate, and spinetoram, the predatory rate was significantly decreased (df = 9/20, F = 64.60, *p* < 0.0001) (Figure 1C). The predation rate of *M. cancestrinnii* significantly decreased after treatment with avermectin, emamectin benzoate, and spinetoram, exhibiting the most pronounced effects (df = 9/20, F = 92.11, *p* < 0.0001) (Figure 1D). In the case of *T. maxillosa,* predatory activity results indicate that the predatory rate has significantly decreased after treatment with avermectin, emamectin benzoate, and spinetoram (df = 9/20, F = 27.03, *p* < 0.0001) (Figure 1E). For *U. insecticeps*, treatment with avermectin, emamectin benzoate, and spinetoram, the predatory rate was significantly decreased (df = 9/20, F = 37.23, *p* < 0.0001) (Figure 1F). While tetraniliprole, triflumezopyram, and chlorantraniliprole had no significant difference from the control treatment for all four species of spiders.

### 3.3. LC_50_ and Risk Assessment of Harmful Insecticides to Predators

During the bioassay experiment, after a 48 h insecticide exposure period, spinetoram remained an extremely high-risk insecticide against *C. lividipennis*, with an LC_50_ value of 3.59 mg/L and a safety factor of 0.02–0.03. While imidacloprid, avermectin, emamectin benzoate, and nitenpyram remained high-risk insecticides with LC50 values of 14.75 mg/L, 3.76 mg/L, 2.5 mg/L, and 30.79 mg/L, respectively, and safety factors of 0.22–0.44, 0.11–0.18, 0.07–0.15, and 0.12–0.15, respectively, against *C. lividipennis*. The toxicity assessment classified nitenpyram as a high-risk insecticide for *P. fuscipes*, with an LC50 value of 52.71 mg/L, and a safety factor of 0.21–0.26.

For *P. pseudoannulata*, avermectin and emamectin benzoate remained high-risk insecticides with LC_50_ values of 5.87 mg/L and 4.86 mg/L, respectively, and the safety factor of 0.17–0.29 and 0.14–0.29, respectively. During risk assessment of insecticides against *M. cancestrinnii*, avermectin, and emamectin benzoate showed high risk response with LC_50_ values of 12.87 mg/L and 9.42 mg/L, respectively, and the safety factors 0.38–0.64 and 0.28–0.56, respectively. Similarly, *T. maxillosa* was highly susceptible to avermectin and emamectin benzoate. Avermectin and emamectin benzoate remained high-risk insecticides with LC_50_ values of 5.98 mg/L and 4.37 mg/L, respectively, and the safety factors of 0.17–0.29 and 0.13–0.26, respectively, to *T. maxillosa*. Both avermectin and emamectin benzoate were classified as extremely high risk for *U. insecticeps*, with LC_50_ values of 0.281 mg/L and 0.16 mg/L, respectively, and the safety factors of 0.008–0.014 and 0.004–0.009, respectively. While spinetoram remained a high-risk insecticide to *U. insecticeps*, with an LC_50_ value of 0.18 mg/L and a safety factor of 0.081–0.101 (Table 4).

## 4. Discussion

In the present study, three novel insecticides—tetraniliprole, triflumezopyram, and chlorantraniliprole—were found to be safe for all six predators (*C. lividipennis*, *P. fuscipes*, *T. maxillosa*, *M. cancestrinnii*, *P. pseudoannulata,* and *U. insecticeps*) during the bioassay experiment and were categorized as harmless. These three insecticides showed no significant negative effects on predation rates. Similar results were reported before. In previous research, tetraniliprole showed safety to *C. lividipennis*, *P. fuscipes*, *Pardosa birmanica,* and *Tetragnatha javana* populations [14,32]. Triflumezopyrim (25 g a.i ha^−1^) exhibited safety to *C. lividipennis*, *P. fuscipes*, *U. insecticeps*, *P. pseudoannulata*, and Tetragnatha sp., which is consistent with our findings [29,34]. Chlorantraniliprole exhibited safety to *C. lividipennis*, *P. fuscipes*, and *Tetragnatha maxillosa* [35,36,37].

In our experimental results, pymetrozine did not cause high mortality in any of six predators; however, it lowered the predatory rate of *C. lividipennis*, *P. fuscipes*, *P. pseudoannulata*, and *T. maxillosa*. Imidacloprid remained harmful against *C. lividipennis* and was graded high risky insecticide. Imidacloprid also lowered the predation rate of *C. lividipennis*, *P. fuscipes*, *M. cancestrinnii*, *T. maxillosa,* and *U. insecticeps*. Nitenpyram showed harmful effects to *C. lividipennis* and *P. fuscipes* and was classified as high risky insecticide, while it lowered the predatory rate of *C. lividipennis*, *P. fuscipes*, *T. maxillosa,* and *U. insecticeps*. Similar results were reported before. In previous studies, nitenpyram had no harmful effects on rice crop spiders [38], but had a negative impact on predatory natural enemies such as *Cyrtorhinus lividipennis* [39]. Imidacloprid showed harmful effects to *C. lividipennis* and *P. fuscipes* [40,41] while being safe to *Hylyphantes graminicola* and *T. maxillosa* [42,43]. Pymetrozine remained safe to rice crop predators, including *C. lividipennis*, *P. fuscipes*, *Pirate subpiraticus,* and *T. maxillosa* [35,37,44].

Emamactin benzoate and avermectin were classified as high risky to *C. lividipennis*, whereas spinetoram was classified as extremely high risky to *C. lividipennis*. For *P. pseudoannulata* and *T. maxillosa,* avermectin and emamactin benzoate remained high risky insecticides. For *M. cancestrinnii* avermectin and emamactin benzoate were classified as medium to high risky insecticides. For *U. insecticeps,* avermectin and emamctin benzoate remained extremely high risky, while spinetoram was classified as extremely high risky to *C. lividipennis* and high risky to *U. insecticeps*. Avermectin, emamactin benzoate, and spinetoram significantly lowered the predation rate of all six predators: *C. lividipennis*, *P. fuscipes*, *T. maxillosa*, *M. cancestrinnii*, *P. pseudoannulata,* and *U. insecticeps*. Similar results were reported before. Studies previously demonstrated that spinetoram was highly toxic to beneficial arthropods, including spiders, *C. lividipennis,* and *P. fuscipes* [45,46] while it showed sublethal effects to *P. pseudoannulata* [47]. Our result shows that emamectin benzoate significantly lowered the predation rate for *P. fuscipes,* which is consistent with previous results. Emamectin benzoate significantly affected *C. lividipennis* and *P. fuscipes*, *Tetragnatha japonica,* and *U. insecticeps* populations in field trials [48,49,50]. Emamectin benzoate, 1/20th of the field concentration, also showed toxic effects to *Pardosa birmanica* by spraying insecticides on Petri dishes under laboratory experiment [51]. A similar result was also observed in avermectin. Avermectin showed high toxicity to *C. lividipennis*, *P. fuscipes*, and *P. pseudoannulata* [29,52] under laboratory experiments, while it lowered the number of *Phidippus audax* and *T. maxillosa* under field trials [53,54]. As avermectin, emamactin benzoate, and spinetoram work on the nervous system, causing permanent paralysis and insect mortality, these insecticides showed high toxicity and lowered the predation rate of predatory natural enemies [55].

Our research indicates that three long-acting insecticides: tetraniliprole, triflumezopyrim, and chlorentraniliprole had a harmless impact on all six predators (*C. lividipennis*, *P. fuscipes*, *T. maxillosa*, *M. cancestrinnii*, *P. pseudoannulata,* and *U. insecticeps*). These three insecticides also showed no indirect toxicity to all six predators, making them suitable for use in rice fields for pest control and compatible with natural enemies. In contrast, nitenpyram, pymetrozine, and imidacloprid showed harmful effects on some predators, while avermectin, emamectin benzoate, and spinetoram exhibited high direct toxicity to all predatory natural enemies and significantly lowered the predation rate of all six predators. It is recommended to reduce and limit the use of these harmful insecticides in rice fields to protect the predatory natural enemies and improve rice production.

## 5. Conclusions

Our findings demonstrate that tetraniliprole, triflumezopyrim, and chlorentraniliprole are non-toxic for all six predators: *Cyrtorhinus lividipennis*, *Paederus fuscipes*, *Tetragnatha maxillosa*, *Mendoza cancestrinnii*, *Pardosa pseudoannulata,* and *Ummeliata insecticeps* at maximum recommended field doses. While Spinetoram, avermectin, emamectin benzoate, pymetrozine, nitenpyram, and imidacloprid showed direct and indirect toxicity effects to predators at maximum recommended field doses. Consequently, our results proved that tetraniliprole, triflumezopyrim, and chlorentraniliprole are compatible with natural enemies and can be used to control rice insect pests. Field trials are needed to further confirm the lethal and sublethal effects of tetraniliprole, triflumezopyrim, and chlorentraniliprole on rice insect pests and their predatory natural enemies.

## Figures and Tables

**Table 1:** Detailed information on insecticides used in this study.

Insecticides	Target Pests	Manufacturers	Concentration (a.i.%)	Field-Recommended Dosage (g a.i ha^−1^)
Triflumezopyrim SC	Rice plant hoppers	Corteva (China) Investment Co., Ltd., Shanghai, China	10	15–24
Pymetrozine WP	Rice plant hoppers	Hebei Veyong Biochemical Co., Ltd., Shijiazhuang, China	50	90–150
Nitenpyram WDG	Rice plant hoppers	Shaanxi Huarong Kaiwei Biological Co., Ltd., Xi’an, China	30	90–112.5
Imidacloprid WP	Rice plant hoppers	Bayer Crop Science (China) Co., Ltd., Hangzhou, China	10	15–30
Spinetoram WDG	Stem borer, Leaf folder	Sino-Agri Leading (Tianjin) Agrochemical Co., Ltd., Tianjin, China	25	15–56.25
Avermectin EC	Stem borer, Leaf folder	Hebei Zhongbao Green Crop Technology Co., Ltd., Langfang, China	5	9–15
Emamectin benzoate WDG	Stem borers	Hebei Zhongbao Green Crop Technology Co., Ltd., Langfang, China	5	7.5–15
Tetraniliprole SC	Stem borers	Bayer Crop Science (China) Co., Ltd., Hangzhou, China	20	21–30
Chlorentraniliprole SC	Stem borers	FMC (China) Investment Co., Ltd., Shanghai, China	20	15–30

**Table 2:** Direct toxicity of nine insecticides to *Cyrtorhinus lividipennis* and *Paederus fuscipes*.

Insecticide	Field Rec. Dose (g a.i ha^−1^)	*Cyrtorhinus lividipennis*		*Paederus fuscipes*	
		Mortality (%) (Mean ± SD)	Toxic Impact (IOBC Category)	Mortality (%) (Mean ± SD)	Toxic Impact (IOBC Category)
Triflumezopyrim	24.00	6.0 ± 5.5	Category 1	4.0 ± 5.5	Category 1
Pymetrozine	150.00	16.0 ± 8.9	Category 1	12.0 ± 8.4	Category 1
Nitenpyram	112.50	100.0 ± 0.0	Category 4	100.0 ± 0.0	Category 4
Imidacloprid	30.00	100.0 ± 0.0	Category 4	12.0 ± 16.4	Category 1
Spinetoram	56.25	100.0 ± 0.0	Category 4	22.0 ± 13.0	Category 1
Avermectin	15.00	100.0 ± 0.0	Category 4	20.0 ± 7.1	Category 1
Emamectin benzoate	15.00	100.0 ± 0.0	Category 4	24.0 ± 11.4	Category 1
Tetraniliprole	30.00	8.0 ± 4.5	Category 1	6.0 ± 8.9	Category 1
Chlorentraniliprole	30.00	10.0 ± 7.1	Category 1	8.0 ± 4.5	Category 1
Control	00.00	2.0 ± 4.47	Category 1	0.0 ± 0.0	Category 1

Note: Insecticides causing mortality < 30% are ranked as harmless, and insecticides caused 100% mortality are ranked as harmful.The same in the Table 3.

**Table 3:** Direct toxicity of nine insecticides to four species of spiders.

Treatment	Field Rec. Dose (g a.i ha^−1^)	*Tetragnatha maxillosa*		*Mendoza cancestrinnii*		*Pardosa pseudoannulata*		*Ummeliata insecticeps*	
		Mortality (%) (Mean ± SD)	Toxic Impact (IOBC Category)	Mortality (%) (Mean ± SD)	Toxic Impact (IOBC Category)	Mortality (%) (Mean ± SD)	Toxic Impact (IOBC Category)	Mortality (%) (Mean ± SD)	Toxic Impact (IOBC Category)
Triflumezopyrim	24.00	4.0 ± 5.5	Category 1	6.0 ± 13.4	Category 1	2.0 ± 4.5	Category 1	8.0 ± 8.4	Category 1
Pymetrozine	150.00	12.0 ± 4.5	Category 1	10.0 ± 7.1	Category 1	12.0 ± 10.9	Category 1	16.0 ± 5.5	Category 1
Nitenpyram	112.50	8.0 ± 8.4	Category 1	8.0 ± 4.5	Category 1	14.0 ± 11.4	Category 1	12.0 ± 4.5	Category 1
Imidacloprid	30.00	10.0 ± 12.2	Category 1	12.0 ± 13.0	Category 1	10.0 ± 7.1	Category 1	14.0 ± 11.4	Category 1
Spinetoram	56.25	30.0 ± 7.1	Category 1	24.0 ± 15.2	Category 1	26.0 ± 8.9	Category 1	100.0 ± 0.0	Category 4
Avermectin	15.00	100.0 ± 0.0	Category 4	100.0 ± 0.0	Category 4	100.0 ± 0.0	Category 4	100.0 ± 0.0	Category 4
Emamectin benzoate	15.00	100.0 ± 0.0	Category 4	100.0 ± 0.0	Category 4	100.0 ± 0.0	Category 4	100.0 ± 0.0	Category 4
Tetraniliprole	30.00	6.0 ± 8.9	Category 1	4.0 ± 8.9	Category 1	8.0 ± 4.5	Category 1	8.0 ± 10.9	Category 1
Chlorentraniliprole	30.00	8.0 ± 13.0	Category 1	8.0 ± 10.9	Category 1	6.0 ± 8.9	Category 1	10.0 ± 7.1	Category 1
Control	00.00	0.0 ± 0.0	Category 1	0.0 ± 0.0	Category 1	0.0 ± 0.0	Category 1	2.0 ± 4.5	Category 1

**Table 4:** LC_50_ values and risk assessment of harmful insecticides on six predators.

Predator’s Name	Insecticide	Slope ± SD	LC_50_ (mg/L)	95% Confidence Interval	X^2^ (df)	*p* Value (Sig.)	Safety Factor	Risk Level
*Cyrtorhinus lividipennis*	Spinetoram	3.28 ± 0.43	3.59	2.95–4.36	6.98 (13)	0.972	0.02–0.03	Extremely high risk
	Imidacloprid	2.99 ± 0.39	14.75	12.02–18.11	7.00 (13)	0.902	0.22–0.44	High risky
	Avermectin	1.94 ± 0.31	3.76	2.66–4.94	11.72 (13)	0.550	0.11–0.18	High risky
	Emamectin benzoate	1.56 ± 0.23	2.50	1.74–3.39	10.98 (16)	0.810	0.07–0.15	High risky
	Nitenpyram	2.20 ± 0.35	30.79	22.21–39.71	3.76 (13)	0.993	0.12–0.15	High risky
*Paederus fuscipes*	Nitenpyram	3.02 ± 0.40	52.71	42.91–64.50	13.01 (13)	0.447	0.21–0.26	High risky
*Pardosa pseudoannulata*	Avermectin	2.09 ± 0.31	5.87	4.50–7.63	10.99 (13)	0.612	0.17–0.29	High risky
	E. Benzoate	1.57 ± 0.23	4.86	3.56–6.66	19.66 (16)	0.24	0.14–0.29	High risky
*Mendoza cancestrinnii*	Avermectin	1.96 ± 0.31	12.87	8.73–19.09	19.46 (13)	0.11	0.38–0.64	Medium to High risk
	E. Benzoate	1.87 ± 0.24	9.42	7.20–12.40	14.35 (16)	0.572	0.28–0.56	Medium to High risk
*Tetragnatha maxillosa*	Avermectin	3.14 ± 0.41	5.98	4.90–7.30	6.43 (13)	0.928	0.17–0.29	High risky
	E. Benzoate	1.73 ± 0.29	4.37	3.09–5.88	9.81 (13)	0.709	0.13–0.26	High risky
*Ummeliata insecticeps*	Avermectin	1.98 ± 0.25	0.28	0.21–0.36	19.06 (16)	0.265	0.008–0.014	Extremely high risk
	E. Benzoate	1.28 ± 0.19	0.16	0.10–0.23	9.37 (16)	0.897	0.004–0.009	Extremely high risk
	Spinetoram	2.28 ± 0.28	10.18	8.02–12.86	4.93 (16)	0.996	0.081–0.101	High risky

Note: *p*-value > 0.05 = the insecticides toxicity data fit the probit model; *p*-value ≤ 0.05 = the insecticides toxicity data do not fit the probit model.

## Data Availability

The original contributions presented in this study are included in the article.
